# Supplementary material for: Association of intraindividual differences in estimated glomerular filtration rates based on cystatin C and creatinine with dementia: A cohort study of the UK Biobank
Source: PLoS One. 2026 Mar 6;21(3):e0344566. doi: 10.1371/journal.pone.0344566 (PMC12965570; doi:10.1371/journal.pone.0344566)
Supplement: S1 File — Flow diagram of analyses. aSensitivity analysis was conducted in this population using multiple imputation to account for missing data on the exposure and covariates or removing low-eGFR participations. S2 Fig. Correlation matrix of kidney function markers. Hexbin plot of the relation between eGFRcr, eGFRcys, and eGFRdiff at baseline. (A) Correlation between eGFRcr and eGFRcys. (B) Correlation between eGFRcr and eGFRdiff. (C) Correlation between eGFRcys and eGFRdiff. S3 Fig. Survival curves and proportional hazards assessment. Kaplan-Meyer survival curves using time-scale and scatter plot of the scaled Schoenfeld residuals for eGFRdiff and all cause dementia(A), Alzheimer’s disease(B) and vascular dementia(C). S4 Fig. Nonlinear dose-response relationships. Dose-response relationship between eGFRratio (A to C) and All cause dementia, Alzheimer’s disease, or Vascular dementia. Restricted cubic spline was used to explore nonlinear associations, with three knots fixed at the quartiles for all smooth curves. Green line representing 95% Confidence interval. The HR was derived using Cox proportional hazard regression. Model were adjusted for eGFRcr_cys, Cho, LDL, education, smoking, drinking, physical activities, Townsend deprivation index (TDI), social isolate, hearing, eyesight, diabetes, hypertension, depression, and obesity. S5 Fig. Sensitivity analyses of eGFR measures. Associations between eGFRdiff or eGFRratio(z-score) and incident dementia. (A) Model 1 were adjusted for Cho, LDL, education, smoking, drinking, physical activities, Townsend deprivation index (TDI), social isolate, hearing, eyesight, diabetes, hypertension, depression, and obesity. (B) Model 2 were further adjusted for eGFRcr_cys. S6 Fig. Risk stratification by optimal eGFRdiff cut-off. Kaplan–Meier curves for incident all-cause dementia according to high- and low-risk groups defined by the optimal cut-off value of eGFRdiff (−8.813) derived from maximally selected rank statistics. S7 Fig. Incremental [file pone.0344566.s001.zip › S5_Table.docx]

**Supplementary Table S5** Associations between difference in cystatin C- and creatinine-based estimated glomerular filtration rate and incident dementia in people with hypertension

| **Outcome and exposure** | **Cases (Rate per  100 person years)** | **Crude hazard ratio  (95%CI)** | **Adjusted hazard ratio  (95%CI)** |
| --- | --- | --- | --- |
| **All cause dementia** |  |  |  |
| Categorical eGFR_diff_ |  |  |  |
| Negative (~-15 ml/min/1.73 m^2^) | 2594/1052.25 (2.47) | 1.0 (ref) | 1.0 (ref) |
| Midrange(~ 15 ml/min/1.73 m^2^) | 4376/2472.36 (1.77) | 0.71 (0.67 to 0.74) | 0.89 (0.85 to 0.94) |
| Positive (> 15 ml/min/1.73 m^2^) | 166/152.07 (1.09) | 0.43 (0.37 to 0.51) | 0.73 (0.63 to 0.86) |
| Continuous eGFR_diff_  (per SD ml/min/1.73m^2^) | | 0.80 (0.78 to 0.82) | 0.92 (0.90 to 0.94) |
| **Alzheimers disease** |  |  |  |
| Categorical eGFR_diff_ |  |  |  |
| Negative (~-15 ml/min/1.73 m^2^) | 1091/1056.47 (1.03) | 1.0 (ref) | 1.0 (ref) |
| Midrange(~ 15 ml/min/1.73 m^2^) | 1987/2478.90 (0.80) | 0.76 (0.71 to 0.82) | 0.93 (0.86 to 1.00) |
| Positive (> 15 ml/min/1.73 m^2^) | 76/152.36 (0.50) | 0.47 (0.37 to 0.60) | 0.78 (0.62 to 0.99) |
| Continuous eGFR_diff_  (per SD ml/min/1.73m^2^) | | 0.83 (0.80 to 0.86) | 0.94 (0.91 to 0.98) |
| **Vascular dementia** |  |  |  |
| Categorical eGFR_diff_ |  |  |  |
| Negative (~-15 ml/min/1.73 m^2^) | 662/1057.70 (0.63) | 1.0 (ref) | 1.0 (ref) |
| Midrange(~ 15 ml/min/1.73 m^2^) | 990/2481.38 (0.40) | 0.63 (0.57 to 0.69) | 0.85 (0.77 to 0.94) |
| Positive (> 15 ml/min/1.73 m^2^) | 34/152.46 (0.22) | 0.35 (0.25 to 0.49) | 0.66 (0.47 to 0.94) |
| Continuous eGFRdiff (per SD ml/min/1.73m^2^) | | 0.75 (0.71 to 0.78) | 0.90 (0.85 to 0.94) |
|  |  |  |  |

**Notes:** Associations between eGFRdiff (quartile group and z-score) and incident dementia. Age-scaled models were adjusted for eGFRcr_cys, Cho, LDL, education, smoking, drinking, physical activities, Townsend deprivation index (TDI), social isolate, hearing, eyesight, and obesity.

**Abbreviations:** eGFRcr_cys, estimated glomerular filtration rate calculated using both creatinine and cystatin C; eGFRdiff, the difference between creatinine-based estimated glomerular filtration rate; TDI, Townsend deprivation index; MET, metabolic equivalent; LDL-C, serum low-density lipoprotein cholesterol; Cho: serum cholesterol; NCDs: Noncommunicable diseases. HR, hazard ratio; CI, confidence interval; AD, Alzheimer’s disease; VaD, vascular dementia.
