# Supplementary material for: Association of intraindividual differences in estimated glomerular filtration rates based on cystatin C and creatinine with dementia: A cohort study of the UK Biobank
Source: PLoS One. 2026 Mar 6;21(3):e0344566. doi: 10.1371/journal.pone.0344566 (PMC12965570; doi:10.1371/journal.pone.0344566)
Supplement: S1 File — Flow diagram of analyses. aSensitivity analysis was conducted in this population using multiple imputation to account for missing data on the exposure and covariates or removing low-eGFR participations. S2 Fig. Correlation matrix of kidney function markers. Hexbin plot of the relation between eGFRcr, eGFRcys, and eGFRdiff at baseline. (A) Correlation between eGFRcr and eGFRcys. (B) Correlation between eGFRcr and eGFRdiff. (C) Correlation between eGFRcys and eGFRdiff. S3 Fig. Survival curves and proportional hazards assessment. Kaplan-Meyer survival curves using time-scale and scatter plot of the scaled Schoenfeld residuals for eGFRdiff and all cause dementia(A), Alzheimer’s disease(B) and vascular dementia(C). S4 Fig. Nonlinear dose-response relationships. Dose-response relationship between eGFRratio (A to C) and All cause dementia, Alzheimer’s disease, or Vascular dementia. Restricted cubic spline was used to explore nonlinear associations, with three knots fixed at the quartiles for all smooth curves. Green line representing 95% Confidence interval. The HR was derived using Cox proportional hazard regression. Model were adjusted for eGFRcr_cys, Cho, LDL, education, smoking, drinking, physical activities, Townsend deprivation index (TDI), social isolate, hearing, eyesight, diabetes, hypertension, depression, and obesity. S5 Fig. Sensitivity analyses of eGFR measures. Associations between eGFRdiff or eGFRratio(z-score) and incident dementia. (A) Model 1 were adjusted for Cho, LDL, education, smoking, drinking, physical activities, Townsend deprivation index (TDI), social isolate, hearing, eyesight, diabetes, hypertension, depression, and obesity. (B) Model 2 were further adjusted for eGFRcr_cys. S6 Fig. Risk stratification by optimal eGFRdiff cut-off. Kaplan–Meier curves for incident all-cause dementia according to high- and low-risk groups defined by the optimal cut-off value of eGFRdiff (−8.813) derived from maximally selected rank statistics. S7 Fig. Incremental [file pone.0344566.s001.zip › S1_Method.docx]

**Supplementary Method: Genetic risk assessment**

APOE genotypes were estimated from 2 single nucleotide polymorphisms (SNP), rs7412 and rs429358. Participants with at least one copy of APOE ε4 were considered APOE ε4 carriers, and others were considered as non-APOE ε4 carriers.

We calculated dementia non-APOE polygenic risk score (PRS) based on a 39-SNP PRS developed by Ebenau et al^[1]^, which predicted AD dementia independently of APOE ε4. Ebenau et al selected those SNPs from genome‐wide association studies (GWAS) for late-onset AD, and derived variant-specific weight from International Genomics of Alzheimer's Project studies. For this analysis, we performed quality controls by excluding one SNP that had minor allele frequency <0.005, leaving 38 SNPs for PRS calculation. The PRS was then divided into quintiles, and participants were categorized into three genetic risk groups: low (lowest PRS quintile), intermediate (PRS quintiles 2 to 4), and high (highest PRS quintile).

**Supplementary Method: Genetic risk assessment**

The optimal cut-off value of the eGFRdiff-based score was determined using maximally selected rank statistics implemented in the surv_cutpoint function of the ‘survminer package, which identifies the threshold that maximizes separation between survival curves. Participants were subsequently categorized into high- and low-risk groups according to this cut-off, and Kaplan–Meier curves were plotted and compared using the log-rank test.

Furthermore, time-dependent receiver operating characteristic (ROC) curves were constructed using the timeROC package to evaluate model discrimination at 5, 10, and 15 years of follow-up^[2]^. Discriminatory performance was assessed for two models: Model 1 including the established UK Biobank Dementia Risk Score (UKBDRS) alone, and Model 2 incorporating eGFRdiff in addition to UKBDRS^[3]^. AUC values were compared descriptively to assess whether eGFRdiff provided incremental discrimination beyond UKBDRS. These analyses were conducted as supplementary evaluations rather than for model development purposes.

SUPPLEMENTARY REFERENCE

^1^ Ebenau JL, van der Lee SJ, Hulsman M, et al. Risk of dementia in APOE ε4 carriers is mitigated by a polygenic risk score. Alzheimers Dement (Amst). 2021;13(1):e12229. doi:10.1002/dad2.12229

^2^ Blanche P, Dartigues JF, Jacqmin-Gadda H. Estimating and Comparing time-dependent areas under receiver operating characteristic curves for censored event times with competing risks. Statistics in Medicine. 2013;32(30):5381-5397. doi:10.1002/sim.5958

^3^ Anatürk M, Patel R, Ebmeier KP, et al. Development and validation of a dementia risk score in the UK Biobank and Whitehall II cohorts. BMJ Mental Health. 2023;26(1):e300719. doi:10.1136/bmjment-2023-300719
