# Supplementary material for: Association of intraindividual differences in estimated glomerular filtration rates based on cystatin C and creatinine with dementia: A cohort study of the UK Biobank
Source: PLoS One. 2026 Mar 6;21(3):e0344566. doi: 10.1371/journal.pone.0344566 (PMC12965570; doi:10.1371/journal.pone.0344566)
Supplement: Attachment — Submitted filename: Response to Reviewers.docx [file pone.0344566.s003.docx]

Dear editor,

We highly appreciate you and all reviewers for reviewing our manuscript ("Association of intraindividual differences in estimated glomerular filtration rates based on cystatin C and creatinine with dementia: a cohort study of the UK Biobank", Submission ID PONE-D-25-60018) and providing the thorough and thoughtful comments. We have seriously considered reviewers’ comments and suggestions, and responded to these comments point-by-point, and revised the manuscript based on group discussion and experts’ advice accordingly. All changes to the manuscript are indicated by using tracked changes. Please see below, in blue, for a point-to-point response to the reviewers’ comments and concerns.

**Point-to-Point Response**

**Responses to the comments of Editor**

**1. Comment:** Please ensure that your manuscript meets PLOS ONE's style requirements, including those for file naming.

**Response:** Thank you for bringing this to our attention. We have carefully cross-checked the revised files against the PLOS ONE style template and adjusted the formatting and file names accordingly. We appreciate your time and effort in overseeing the review process. Should any further modifications be needed, we would be happy to make them promptly.

**2. Comment: Please include your amended Funding Statement within your cover letter. We will change the online submission form on your behalf.**

**Response:** Thank you for highlighting the need to amend the Funding Statement. We have carefully revised the statement in both the manuscript and the cover letter to ensure consistency and completeness. The updated Funding Statement now reads as follows:” This work was supported by the National Natural Science Foundation of China (grant No. 82273730 to YY), the Shanghai Municipal Natural Science Foundation (grant No. 22ZR1414900 to YY), and the Three-Year Public Health Action Plan of Shanghai (grants GWVI-11.2-XD10 and GWVI-11.1-01 to YY). The funders had no role in study design, data collection and analysis, decision to publish, or preparation of the manuscript. There was no additional external funding received for this study.”

**3. Comment:** All PLOS journals now require all data underlying the findings described in their manuscript to be freely available to other researchers, either a. In a public repository, b. Within the manuscript itself, or c. Uploaded as supplementary information.

**Response:** Thank you for highlighting PLOS’ open data policy. We fully support the principles of transparency and reproducibility.

All statistical analyses were performed with previously published R packages. We have now Added the exact package names and versions in the Methods (page 11, lines 193) and uploaded the complete analysis workflow as Supplementary File.

Regarding the individual-level data: These are available only through a formal application to UK Biobank (https://www.ukbiobank.ac.uk). Public deposition is explicitly prohibited by the UK Biobank Material Transfer Agreement and by Research Ethics Committee approval 16/NW/0274. We therefore request an exemption from mandatory public deposition, following the procedure outlined in the PLOS data policy. Comparable exemptions have been granted in recent papers published in BMJ, Nature Medicine, and Nature Communications, confirming that this approach is consistent with peer-reviewed practice. Any researcher can reproduce our findings by Applying for UK Biobank access.

We have inserted the following Data Availability statement in the manuscript (page 28): “Data are available from UK Biobank upon application (www.ukbiobank.ac.uk). Public deposition of individual-level data is prohibited under the UK Biobank Material Transfer Agreement and Research Ethics Committee approval (16/NW/0274). The analysis code and variable lists are provided in Supplementary File S1. Researchers wishing to replicate the analyses can apply for access through the standard Access Management System.”
” Please let us know if any additional information is required.

**4. Comment:** PLOS requires an ORCID iD for the corresponding author in Editorial Manager on papers submitted after December 6th, 2016. Please ensure that you have an ORCID iD and that it is validated in Editorial.

**Response:** Thank you for your reminder. We will update the corresponding author's validated ORCID iD in Editorial Manager as required.

**5. Comment:** Your ethics statement should only appear in the Methods section of your manuscript. If your ethics statement is written in any section besides the Methods, please delete it from any other section.

**Response:** Thank you for your guidance. We have removed any duplicate ethics statements from sections other than the Methods and ensured that the complete ethics statement appears only in the Methods section as required.

**6. Comment:** Please include captions for your Supporting Information files at the end of your manuscript, and update any in-text citations to match accordingly.

**Response:** Thank you for this suggestion. We have now added a "Supporting Information" section at the end of the manuscript.

**7. Comment:** Please upload a new copy of Figures 1, S3 and S4 as the detail is not clear.

**Response:** Thank you for your comment. We have uploaded all high-resolution images and re-added them to the document.

**Responses to the comments of Reviewer 1**

**1. Comment:** Are there additional biochemical or molecular data evaluated in the study that were not presented in the manuscript.

**Response:** Thank you for this insightful suggestion.

We fully agree that integrating proteomic and metabolomic data could deepen mechanistic insight.

The UK Biobank has indeed generated plasma proteomics (~1,500 aptamers) and targeted metabolomics (~150 absolutely quantified lipids/metabolites). However, the present manuscript is designed as a population-level epidemiologic study whose primary aim is to quantify the association between lower eGFRdiff and dementia risk. For two reasons we elected to defer an in-depth molecular dissection to a separate investigation:

1. Temporal overlap: Because the -omics measures are drawn from the same baseline visit as eGFRdiff, they cannot be treated as mediators without violating the temporal ordering required for formal mediation analysis.
2. Sample-size constraints: Proteomics and metabolomics are currently available for only ~12 000 and ~6 000 participants, respectively. Restricting the primary analysis to these subsets would reduce statistical precision by >90 % without a corresponding gain in mechanistic clarity.

To address this concern, we have now added the following explicit statement to the Discussion (page 2w lines 369–379):

“Future investigations that obtain repeated -omics measurements and employ time-resolved mediation models are needed to determine whether systemic inflammation, endothelial dysfunction, or metabolic reprogramming transmit the effect of early kidney dysfunction on dementia risk.”
We hope this clarification underscores both our awareness of the molecular data and our intention to pursue mechanism-focused questions in future work.

**2. Comment:** Is the genetic component considered in patients with pre-existing conditions?

**Response:** We thank the reviewer for this insightful comment.

In our original manuscript, we conducted stratified sensitivity analyses according to APOE ε4 carrier status and dementia-specific polygenic risk score (PRS) strata (Fig 2), which yielded consistent effect estimates across genetic risk groups.

To further evaluate the possibility of genetic confounding, in addition to adjustment for APOE ε4 and dementia-PRS, we performed a genome-wide association study of eGFRdiff in approximately 450,000 UK Biobank participants and estimated genetic correlations using LD-score regression (LDSC) with all-cause dementia, Alzheimer’s disease, and vascular dementia. The resulting rg estimates were close to zero (ACD: rg = 0.0380, SE = 0.0270, P = 0.150; AD: rg = 0.0221, SE = 0.0386, P = 0.566; VaD: rg = −0.0394, SE = 0.0591, P = 0.505), and the 95% confidence intervals did not indicate moderate or strong genetic correlations.

| **Trait 1** | **Trait 2** | **rg** | **SE** | **P value** |
| --- | --- | --- | --- | --- |
| eGFRdiff | All-cause dementia | 0.0380 | 0.0270 | 0.150 |
| eGFRdiff | Alzheimer’s’ disease | 0.0221 | 0.0386 | 0.566 |
| eGFRdiff | Vascular dementia | -0.0394 | 0.0591 | 0.505 |

In response to the reviewer’s specific concern regarding patients with pre-existing conditions, we additionally repeated the primary analyses within subgroups of participants with major pre-existing diseases, including diabetes, hypertension, and depression. The effect estimates were comparable to those observed in the full cohort, indicating that the association between eGFRdiff and dementia risk is preserved even among clinically vulnerable populations. These subgroup analyses have now been included as sensitivity analyses and are presented in S4-S6 Tables.

Although these disease-specific subgroup analyses were not stratified by genetic risk, the consistency of associations across genetic risk strata, the absence of detectable shared heritability, and the robustness of results in participants with pre-existing conditions together suggest that genetic confounding is unlikely to explain the observed eGFRdiff–dementia association. Given this convergence of evidence, extending the model to additional PRSs would be unlikely to substantially alter the inference, and we therefore retained the current analytical framework.

We appreciate the reviewer’s suggestion to examine this issue in greater depth, which motivated these additional sensitivity analyses.

**3. Comment:** What should be the optimal eGFRdiff threshold for screening?

**Response:** We appreciate this critical inquiry from the reviewer. To determine a potential screening threshold, we applied maximally selected rank statistics through the surv_cutpoint function in R. This statistical method was selected for its robustness in identifying optimal cut-offs that maximize the discrimination between survival curves in our dataset. The identified cut-off value at −8.813 facilitated the classification of participants into high- and low-risk groups, revealing significantly different cumulative incidences of dementia (log-rank p < 0.001; S6 Fig). However, the clinical relevance of such a distinction, in terms of effect size and impact on patient management, remains to be fully elucidated.

We stress that the derived cut-off is intended for exploratory risk stratification and not for immediate clinical application as a definitive screening tool. Further, our analysis indicated that while eGFRdiff adds modestly to the discriminative power of the established UK Biobank Dementia Risk Score (UKBDRS), its primary utility may be in highlighting systemic vulnerabilities related to neurodegeneration (S7 Fig).

Consequently, we present these findings as preliminary. Definitive recommendations for a screening threshold based on eGFRdiff would require further studies focused on external validation, detailed cost-benefit evaluations, and exploration of the cut-off's impact in diverse clinical settings. We have now added the corresponding limitation to the revised manuscript. Further study is warranted to ensure that any proposed screening thresholds are both scientifically robust and clinically valuable.

**Responses to the comments of Reviewer 2**

**1. Comment:** eGFRdiff is strongly influenced by non-renal determinants. The authors should discuss this in the discussion.

**Response:** Thank you for the comment and suggestion.

We agree with the reviewer that eGFRdiff is strongly influenced by non-renal determinants. We have now explicitly discussed that eGFRdiff reflects systemic conditions such as sarcopenia, inflammation, and metabolic dysregulation rather than kidney function alone, and that it should be interpreted as an integrated marker of overall health and biological aging. We also added this point to the limitations section and clarified that the observed associations do not imply direct renal causality (Discussion, pages 16, line 295).

**2. Comment:** Hospital-based dementia diagnoses have substantial misclassification, particularly under-ascertainment in early dementia.

**Response:** Thank you for raising this important methodological issue. We fully agree that there may be misclassification in foundational hospital diagnoses, especially in the under-recognition of early-stage dementia.

To assess the impact of this potential misclassification on our main findings, we conducted additional sensitivity analyses. Specifically, we used a method with a 5-year lag-time: patients diagnosed with dementia within 5 years post-exposure assessment were considered as possibly having had dementia at baseline, and these individuals were excluded from the analysis. This approach is also used in similar studies to exclude reverse causality. This method helps reduce the risk of erroneously including individuals who were in the early stages of dementia at baseline but not yet clinically recognized[1].

The results show that, after this conservative exclusion, our main associations remained consistent and the effect estimates were similar to the primary analysis. The results of this sensitivity analysis suggest that, even considering the underdiagnosis of early-stage dementia and potential misclassifications, our main findings remain robust and reliable. We have added a detailed description of this additional analysis in the Methods section (line 185, page 10) and Results section (line 269 page 16) of the revised manuscript.

We appreciate your insightful comment, which has helped us further strengthen the validity of our findings

**3. Comment:** Many modifiable dementia risk factors were included, important confounders remain unaccounted for: cardiovascular disease history and medication use. Given that eGFRdiff correlates with frailty, chronic inflammation, and general health status, residual confounding likely remains.

**Response:** We thank the reviewer for highlighting the possibility of residual confounding. To evaluate how robust our findings are, we now report two additional analyses.

First, we calculated the E-value for the association most central to our hypothesis: “eGFRdiff per 1-SD increase and incident all-cause dementia”. The observed point estimate was HR = 0.92 (95 % CI 0.90–0.94), yielding an E-value of 1.43. An unmeasured confounder (e.g., cardiovascular disease history or medication use) would therefore need to be associated with both eGFRdiff and dementia by a risk-ratio of ≥ 1.43 each, simultaneously, to fully nullify the observed association. This magnitude is larger than the effect sizes typically reported for individual cardiovascular risk factors, suggesting that residual confounding alone is unlikely to explain our result.

Second, to examine whether eGFRdiff merely reflects generic ill health, we conducted a negative-outcome analysis using ICD-10 codes S00–T35 & T66–T78 (injuries, poisoning, burns, etc.)—conditions that have no established biological link with kidney function. Neither categorical nor continuous eGFRdiff was associated with these outcomes (per-SD RR = 1.00, 95 % CI 0.99–1.01), arguing against a non-specific frailty artefact.

We have added these analyses (new S7 Table) to the revised manuscript. We appreciate the reviewer’s suggestions, which have helped us clarify the robustness of our findings.

**4. Comment:** Brain MRI was performed years after baseline measurements for many participants. The manuscript needs clearer explanation of this.

**Response:** We thank the reviewer for raising this important point regarding the temporal relationship between baseline measurements and MRI acquisition.

We have now clarified this in the Methods section: "MRI was acquired a median of 4.7 years (IQR 3.9–5.5) after baseline, ensuring that the exposure (eGFRdiff) temporally preceded the imaging outcomes."

Rather than a limitation, this time interval strengthens the temporal inference of our study. The substantial lag between exposure assessment and MRI acquisition (median 4.7 years) makes reverse causation—whereby subclinical structural brain changes could influence kidney function—highly unlikely. We have added this point to the method (page 10, line 188) and discussion (page 17, line 284).

**5. Comment:** Several statements in the Discussion and Conclusion suggest that eGFRdiff may be an early biomarker of dementia. Given the observational design, this is too causal.

**Response:** Thank you for this important comment. We fully agree that our cross-sectional and prospective analyses are observational and thus cannot establish causality. In the revised manuscript we have:

Replaced all causal language such as “may serve as”, “is an early biomarker”, “indicates”, “predicts” with associative wording (“was associated with”, “might be considered as a potential indicator”, “could support its utility as a hypothesis-generating marker”, etc.).

Added an explicit sentence in both the Discussion (paragraph 1) and the Conclusion: “Because of the observational nature of this study, these findings support but do not prove a causal role of eGFRdiff in dementia pathogenesis.”

We believe these revisions adequately temper the interpretation and avoid causal over-statement.

**Responses to the comments of Reviewer 3**

**1. Comment:** The weight of the patients was set at 64.3kg. Was this data derived from the clinical trial or other sources? It needs to be elaborated.
This figure seems close to the average weight of Chinese men, but lung cancer patients seem to be expected to weigh less than the general population.

**Response:** We sincerely thank the reviewer for raising this important concern. The reviewer is absolutely correct that GFR declines with age and that chronic kidney disease (CKD) is itself a recognized risk factor for dementia. We fully acknowledge that disentangling the effects of kidney function from our findings is critical, and we have attempted to address this through several methodological approaches, though we welcome any further suggestions the reviewer may have.

First, we excluded participants with severely impaired kidney function at baseline (eGFR <30 mL/min/1.73 m², calculated using either creatinine or cystatin C) to minimize the influence of advanced kidney disease. Second, we adjusted for eGFRcr_cys in all our main models to account for overall kidney function. Third, we conducted a sensitivity analysis excluding participants with eGFR <60 mL/min/1.73 m², given the potential unreliability of eGFR measurements in this range, and the results remained consistent with our main findings (S2 Table).

We acknowledge that these approaches may not fully eliminate the complex relationships between aging, kidney function, and dementia risk. However, we observed that eGFRdiff remained significantly associated with dementia outcomes even after adjusting for eGFRcr_cys, which we hope suggests that this measure may capture information beyond kidney function alone—possibly reflecting factors such as muscle mass, inflammation, or vascular health. We recognize that further research is needed to fully elucidate these mechanisms.

We have revised the manuscript to clarify these methodological considerations and would welcome any further suggestions from the reviewer.

**2. Comment:** What were the serum urea values? Did a differential diagnosis of uremic encephalopathy or uremic dementia make?

**Response:** Thank you for the comment. We appreciate your concern regarding the potential confounding effect of uremic encephalopathy or uremic dementia.

We would like to clarify that our study primarily focused on the general population without severe kidney dysfunction. The dementia outcomes in our study were algorithmically defined by the UK Biobank Outcome Adjudication Group, which used hospital inpatient records and death registers based on ICD codes. This approach inherently captures clinically diagnosed dementia cases in community-dwelling individuals and does not specifically identify or exclude "uremic dementia" as a separate entity.

To address your concern about potential confounding by serum urea levels, we have conducted an additional sensitivity analysis further adjusting for serum urea as a covariate. The results remained consistent with our main findings (see table below), suggesting that serum urea did not substantially confound the observed associations between eGFRdiff and dementia risk.

**Table: Association between difference in cystatin C- and creatinine-based estimated glomerular filtration rate and incident dementia**

| **Outcome and exposure** | **Cases/Follow-up(Rate per 100 person years)** | **Crude hazard ratio  (95%CI)** | **Adjusted hazard ratio  (95%CI)** |
| --- | --- | --- | --- |
| **All cause dementia** |  |  |  |
| Categorical eGFR_diff_ |  |  |  |
| Negative (~-15 ml/min/1.73m2) | 765/365.99 (2.09) | 1.0 (ref) | 1.0 (ref) |
| Midrange(~ 15 ml/min/1.73m2) | 1150/945.59 (1.22) | 0.57 (0.52 to 0.63) | 0.82 (0.75 to 0.91) |
| Positive (> 15 ml/min/1.73m2) | 41/69.84 (0.59) | 0.28 (0.20 to 0.38) | 0.59 (0.43 to 0.81) |
| Continuous eGFR_diff_  (per SD ml/min/1.73m2) | | 0.71 (0.68 to 0.74) | 0.88 (0.83 to 0.93) |
| **Alzheimers disease** |  |  |  |
| Categorical eGFR_diff_ |  |  |  |
| Negative (~-15 ml/min/1.73m2) | 312/367.37 (0.85) | 1.0 (ref) | 1.0 (ref) |
| Midrange(~ 15 ml/min/1.73m2) | 482/947.61 (0.51) | 0.59 (0.51 to 0.68) | 0.82 (0.70 to 0.95) |
| Positive (> 15 ml/min/1.73m2) | 22/69.89 (0.31) | 0.36 (0.24 to 0.56) | 0.78 (0.50 to 1.22) |
| Continuous eGFR_diff_  (per SD ml/min/1.73m2) | | 0.73 (0.68 to 0.78) | 0.88 (0.81 to 0.96) |
| **Vascular dementia** |  |  |  |
| Categorical eGFR_diff_ |  |  |  |
| Negative (~-15 ml/min/1.73m2) | 183/367.72 (0.50) | 1.0 (ref) | 1.0 (ref) |
| Midrange(~ 15 ml/min/1.73m2) | 258/948.24 (0.27) | 0.54 (0.45 to 0.65) | 0.84 (0.68 to 1.03) |
| Positive (> 15 ml/min/1.73m2) | 11/69.92 (0.16) | 0.31 (0.17 to 0.57) | 0.76 (0.41 to 1.40) |
| Continuous eGFR_diff_ (per SD ml/min/1.73m2) | | 0.71 (0.65 to 0.78) | 0.94 (0.84 to 1.05) |
|  |  |  |  |

Associations between eGFR_diff_ and incident dementia after imputation of missing covariates. Age-scaled models were adjusted for eGFR_cr_cys_, Cho, LDL, education, smoking, drinking, physical activities, Townsend deprivation index (TDI), social isolate, hearing, eyesight, diabetes, hypertension, depression, obesity and serum urea. HR, hazard ratio; CI, confidence interval; AD, Alzheimer’s disease; VaD, vascular dementia.

Due to space constraints and because the main conclusions remained unchanged, we have not included this sensitivity analysis in the revised manuscript. However, we would be happy to incorporate it if you deem it necessary.

**3. Comment:** What were the kidney imaging results?

**Response:** Thank you for the comment.

We would like to clarify that kidney imaging was not included in our approved UK Biobank data application (Application ID: 98410). The UK Biobank imaging enhancement primarily focuses on brain MRI, with no routine kidney imaging collection available in the baseline assessment.

Our study utilized serum biomarkers (creatinine and cystatin C) to estimate kidney function and examined their intraindividual difference as a potential indicator of overall health status and dementia risk. The eGFRdiff has been validated in previous studies as a clinically relevant marker associated with various adverse outcomes, including frailty, cardiovascular events, and mortality, independent of direct kidney structural assessment.

We agree that incorporating kidney imaging data (such as ultrasound or CT assessments of kidney size, cortical thickness, or nephrosclerosis) would provide valuable insights into the structural correlates of eGFR discrepancies and their relationship with dementia. We propose that future studies with access to such data investigate whether kidney structural abnormalities mediate or modify the association between eGFRdiff and dementia outcomes.

**4. Comment:** What were the kidney imaging results?

**Response:** Thank you for the comment. We appreciate the reviewer's suggestion regarding the assessment of kidney failure duration and treatment compliance monitoring (including phosphate [P], calcium [Ca], parathyroid hormone [PTH], and hemoglobin [Hgb] values). However, these detailed clinical parameters related to kidney failure management are not available in the UK Biobank dataset that we accessed. The UK Biobank provides baseline serum creatinine and cystatin C measurements for eGFR estimation, but does not include longitudinal data on kidney failure progression or treatment-related biochemical markers such as P, Ca, PTH, and Hgb. We agree that incorporating these variables would provide valuable insights into the relationship between kidney disease management and dementia risk, and we suggest that future studies with access to comprehensive renal registry or electronic health record data consider these important factors.

**Responses to the comments of Reviewer 4**

**1. Comment:** While the authors propose several mechanisms (sarcopenia pathway for AD, microvascular complications for VaD), the biological basis for why eGFRdiff predicts dementia remains speculative. The manuscript would benefit from a more coherent mechanistic framework or acknowledgment of this limitation upfront.

**Response:** Thank you for this thoughtful comment. We acknowledge that the proposed mechanisms linking eGFRdiff to dementia—specifically the sarcopenia pathway for Alzheimer's disease and microvascular complications for vascular dementia—remain hypothetical and warrant further experimental validation. As an observational cohort study, our analysis is inherently limited in establishing causality or dissecting underlying biological pathways.

We have now explicitly addressed this limitation in the revised manuscript. In the Discussion section (Limitations subsection), we have added the following statement: "The biological mechanisms underlying the association between eGFRdiff and dementia remain speculative. While we hypothesize potential pathways involving sarcopenia and microvascular dysfunction, these proposed mechanisms are based on indirect evidence and require further mechanistic studies to confirm." Additionally, in the Interpretation of the findings subsection, we have softened our mechanistic claims by replacing definitive language with more cautious phrasing (e.g., "may serve as" instead of "serve as," "potentially influences" instead of "influences") to better reflect the observational nature of our data.

We agree that a fully elucidated mechanistic framework is beyond the scope of this epidemiological study. Our primary contribution lies in identifying eGFRdiff as a potentially accessible early biomarker for dementia risk, which we hope will stimulate future research into its biological underpinnings.

**2. Comment:** The cohort is 94.6% white and from the UK. The significant interaction with ethnicity (particularly for VaD) raises questions about the applicability of these findings to diverse populations. This limitation needs more prominent discussion.

**Response:** We sincerely appreciate this critical observation, which highlights an important limitation of our study that we had not adequately emphasized in the original manuscript.

We fully acknowledge that the ethnic homogeneity of our cohort (94.6% White) significantly constrains the generalizability of our findings, particularly given the significant ethnicity interaction observed for vascular dementia (VaD). On the significant ethnicity interaction for VaD, we agree this finding requires careful interpretation. Non-White participants comprised only 5.4% of the sample, severely limiting statistical power for reliable subgroup estimates. This interaction may reflect: (i) genuine biological heterogeneity in vascular risk patterns across ethnic groups, (ii) chance findings due to small numbers, or (iii) residual confounding. Given these uncertainties, we have added explicit caveats cautioning against over-interpretation, while retaining the data with appropriate qualifications. We believe transparency about such limitations serves the scientific record better than selective presentation.

We fully acknowledge that findings from UK Biobank require validation in diverse cohorts before clinical translation. At the same time, this resource has generated foundational evidence for numerous clinical associations later validated across populations. Our study provides the first large-scale investigation of eGFRdiff in relation to dementia risk, neuroimaging markers, and cognitive function. We explicitly frame these findings as hypothesis-generating and have added a prominent call for multi-ethnic replication studies to confirm and extend our observations.

Specific revisions to the manuscript:

- We have substantially expanded the Limitations section to discuss ethnic homogeneity as a major constraint on generalizability (Page 26, Lines 417)
- We have added explicit caveats in the Clinical Implications section, emphasizing that our findings establish proof-of-concept within a European-ancestry population and require validation in diverse cohorts before clinical application (Page 24, Lines 384)
- We have tempered our Conclusions to frame the study as providing foundational evidence for hypothesis generation rather than universally applicable clinical guidance

**3. Comment:** While the authors suggest eGFRdiff could serve as an early biomarker, the manuscript lacks practical guidance on implementation. What eGFRdiff threshold would trigger clinical action? How does its predictive value compare to existing risk scores?

**Response:** Thank you for the comment. We have ensured all abbreviations are defined on first mention.

We appreciate this critical inquiry regarding clinical implementation. To determine a potential threshold for clinical action, we applied maximally selected rank statistics through the surv_cutpoint function in R. This statistical method was selected for its robustness in identifying optimal cut-offs that maximize the discrimination between survival curves in our dataset. The identified cut-off value at −8.813 facilitated the classification of participants into high- and low-risk groups, revealing significantly different cumulative incidences of dementia (log-rank p < 0.001; S6 Fig). However, the clinical relevance of such a distinction, in terms of effect size and impact on patient management, remains to be fully elucidated.

We stress that the derived cut-off is intended for exploratory risk stratification and not for immediate clinical application as a definitive decision tool. Regarding its predictive value compared to existing risk scores, our analysis indicated that while eGFRdiff adds modestly to the discriminative power of the established UK Biobank Dementia Risk Score (UKBDRS), its primary utility may be in highlighting systemic vulnerabilities related to neurodegeneration (S7 Fig).

Consequently, we present these findings as preliminary. Definitive recommendations for a clinical action threshold based on eGFRdiff would require further studies focused on external validation, detailed cost-benefit evaluations, and exploration of the cut-off's impact in diverse clinical settings. We have now added the corresponding limitation to the revised manuscript. Further study is warranted to ensure that any proposed thresholds are both scientifically robust and clinically valuable.

**4. Comment:** Relying solely on hospital records and death certificates likely underestimates dementia incidence, particularly for mild cases. This could bias the associations, especially if detection bias differs across eGFRdiff groups.

**Response:** Thank you for raising this important concern regarding potential detection bias across eGFRdiff groups. We agree that relying on hospital records and death certificates may underestimate dementia incidence, particularly for mild cases.

To address this concern, we conducted two sensitivity analyses:

First, following your suggestion regarding differential detection, we performed an analysis excluding individuals with any eGFR <60 at baseline. This exclusion helps ensure that our comparisons are not confounded by differential healthcare utilization or diagnostic intensity between those with and without chronic kidney disease, who may have inherently different patterns of medical surveillance. The results remained consistent with our main findings.

Second, to further mitigate concerns about under-ascertainment of early-stage dementia, we applied a 5-year lag-time approach where patients diagnosed with dementia within 5 years post-exposure assessment were considered as possibly having had dementia at baseline and were excluded[1]. This conservative approach addresses the concern that mild cases in early stages may not have been captured in hospital records. The results demonstrated that our main associations remained robust.

We believe these complementary approaches—addressing both differential detection across risk groups and temporal misclassification—strengthen the validity of our findings. We have added detailed descriptions of these additional analyses in the Methods section (page 10) and Results section (page 16) of the revised manuscript.

We appreciate your insightful comment, which has helped us further strengthen the methodological rigor of our study.

Finally, we read through the entire text and made language improvements. Thank you again for your review and valuable comments.

Best regards,

Sincerely,

Xueying Zheng

On behalf of all authors

Department of Biostatistics, School of Public Health, and The Key Laboratory of Public Health Safety of Ministry of Education, Fudan University, Shanghai, China

Email: xyzheng@fudan.edu.cn

Tel: +86 21 54237625

**REFERENCES**

1.	Alders P, Kok A, Zutphen EM van, Claassen JAHR, Deeg DJH. The effect of sleep disturbances on the incidence of dementia for varying lag times. The Journal of Prevention of Alzheimer’s Disease. 2025;12: 100024. doi:https://doi.org/10.1016/j.tjpad.2024.100024
